# Does the Level of Motivation of Physical Education Teachers Matter in Terms of Job Satisfaction and Emotional Exhaustion? A Person-Centered Examination Based on Self-Determination Theory

**DOI:** 10.3390/ijerph16162839

**Published:** 2019-08-08

**Authors:** Ángel Abós, Leen Haerens, Javier Sevil-Serrano, Sofie Morbée, José Antonio Julián, Luis García-González

**Affiliations:** 1 Department of Didactics of the Musical, Plastic and Corporal Expression, Faculty of Health and Sport Sciences, University of Zaragoza, 22001 Huesca, Spain; 2 Department of Movement and Sports Sciences, Faculty of Medicine and Health Sciences, Ghent University, 9000 Ghent, Belgium; 3 Department of Developmental, Personality, and Social Psychology, Faculty of Psychology and Educational Sciences, Ghent University, 9000 Ghent, Belgium; 4 Department of Didactics of the Musical, Plastic and Corporal Expression, Faculty of Social Sciences and Humanities, University of Zaragoza, 22003 Huesca, Spain

**Keywords:** physical education, teachers’ motivation, person-centered approach, job satisfaction, emotional exhaustion, self-determination theory

## Abstract

Grounded in self-determination theory (SDT), prior research has demonstrated that physical education (PE) teachers may have different reasons to engage in teaching. Although some person-centered studies have identified varied motivational profiles in PE teachers, none of these studies have included the three forms of motivation (i.e., autonomous motivation, controlled motivation, and amotivation). This study aims to identify teachers’ motivational profiles, using the three forms of motivation. Moreover, differences between the obtained profiles in terms of job satisfaction and emotional exhaustion were examined. A sample of 107 primary school PE teachers participated. Four distinct motivational profiles were identified: “relatively amotivated”, “somewhat motivated”, “autonomous-controlled motivated”, and “relatively autonomously motivated”. Results showed that the predominantly autonomously motivated PE teachers reported the most adaptive pattern of outcomes. Although PE teachers from the “relatively autonomously motivated” group did not differ in terms of job satisfaction when compared to those in the “autonomous-controlled motivated” group, the former displayed lower values of emotional exhaustion. These findings support SDT in that more motivation is not necessarily better if this additional motivation comes from controlled reasons. These results could raise awareness among school stakeholders about the importance of increasing PE teachers’ autonomous motivation.

## 1. Introduction

Self-determination theory (SDT) [1,2] suggests that physical education (PE) teachers may make an effort in their jobs for different reasons. These reasons may be more or less self-determined, and thus, can differently impact their psychological functioning at work [3,4]. By way of introduction, these reasons or motivations for teaching may be described as autonomous (e.g., PE teaching is valuable and a passion), controlled (e.g., PE teaching is an obligation), and amotivated (e.g., PE teaching is useless). Most studies to date have adopted a variable-centered approach to examine the link between different forms of motivation and psychological functioning in PE teachers [4,5]. Although this approach is useful to examine the association between different variables at the group level, it does not take into account the fact that PE teachers may combine distinct reasons for doing their jobs, as is the case with a person-centered approach. Two person-centered studies have shown that PE teachers may combine autonomous and controlled reasons for making an effort at work [3,6]. The present study extends past research on PE teachers by identifying motivational profiles, using not only autonomous and controlled motivation, but also amotivation. This is a topic that has not been explored among PE teachers. The second essential question in this study is how these within-person motivational profiles relate to PE teachers’ psychological functioning, particularly to their job satisfaction and emotional exhaustion.

### 1.1. Overview of Physical Education Teachers’ Context

It is well known that PE teachers play a key role in promoting health-related behaviors among youth [7,8]. At the same time, international research has highlighted that PE often occupies an inferior position within the school context, and in society in general [9], and therefore has become a subject considered to have low importance [10]. Other studies have noted that PE teachers usually have to operate in poor sports facilities with a lack of sports material and equipment [11], which sometimes even has to be shared with other PE teachers [12]. Moreover, PE lessons are usually held in sports facilities, such as gyms or playgrounds, where children can release their extra energy [13]. Consequently, PE teachers are sometimes faced with challenging class management issues, investing a large amount of energy in controlling students’ misbehavior [11]. Finally, the low number of PE hours in the curriculum has generated teacher dissatisfaction in recent decades [9,12]. This lack of recognition, together with the challenges inherent to PE teaching, may affect teachers’ job satisfaction [14]. Likewise, many PE teachers characterize their jobs as demanding and stressful [15], which may also trigger negative feelings such as frustration or emotional exhaustion [4].

### 1.2. Physical Education Teachers’ Motivation from a Self-Determination Theory Perspective

Since the 1980s, SDT [1,2] has been a widely-used theoretical framework in a variety of areas (i.e., education, sport, work, etc.) to explain why people engage in an activity. In the teaching context, and particularly for PE teachers, the number of studies examining teachers’ motivation for teaching has started to increase in recent years [5,6,16]. Central to SDT is the distinction between qualitatively different types of motivation that are situated on a motivational continuum, which ranges from highly self-determined or internalized forms of motivation (i.e., autonomous motivation) to less self-determined forms of motivation (i.e., controlled motivation), and even amotivation [17,18].

Autonomous motivation involves two types of regulations (i.e., intrinsic motivation and identified regulation), and is characterized by an internal sense of volition and self-acceptance towards an activity [18,19]. Intrinsic motivation refers to the inherent enjoyment of conducting an activity. For example, an intrinsically motivated PE teacher takes pleasure in teaching new PE-related learnings and skills to students. Identified regulation originates from the identification of the relevance and applicability of certain activities. To illustrate, a PE teacher may value the relevance of promoting students’ health-related behaviors. Controlled motivation, located in the middle of the motivational continuum, also involves the two forms of motivation (i.e., introjected regulation and external regulation) and is typified by internal and external feelings of pressure or coercion to engage in certain activities [18,19]. Introjected regulation may arise from internal pressures such as a desire to feel better about oneself or to not feel guilty. For instance, PE teachers may prepare their lessons because of their interest in proving they are good teachers, or to avoid feeling bad about themselves. External regulation may originate from external pressures, such as the desire to perceive rewards or to avoid disapproval and criticism. PE teachers may put effort into their jobs to obtain their colleagues’ recognition, or to avoid a negative evaluation from the school board. Amotivation, which is characterized by a complete absence of self-determination, is located on the opposite side of autonomous motivation in the motivational continuum [18,19]. Amotivated individuals do not believe that their efforts will result in a desired outcome and, therefore, they lose their motivation to put time, effort, and energy into their teaching tasks. For example, PE teachers may think that, no matter what they do, their efforts will not contribute to students’ learning.

### 1.3. Link between Motivation and Psychological Functioning among PE Teachers

It is widely known that happy teachers have better job performance [14,20]. Job satisfaction, given its association with self-determined motivation and job effectiveness, plays a pivotal role in helping PE teachers to thrive within the arduous school environment [14]. In the present study, we rely on the global definition of job satisfaction, which refers to general satisfaction with work as a whole [20]. Together with job satisfaction, emotional exhaustion may broadly represent the psychological functioning of PE teachers [21]. Emotional exhaustion is defined as the state of depletion of one’s emotional resources, and feelings of fatigue at work. Along with depersonalization (i.e., feeling apathetic towards work or the people at work), and reduced personal accomplishment (i.e., feeling less effective at work), emotional exhaustion encompasses the classic definition of burnout [22]. Because emotional exhaustion is widely considered as the key, initial, and central component of burnout [23], this study focuses on this burnout dimension.

In this sense, SDT also offers well-directed predictions about how autonomous motivation, controlled motivation, and amotivation may determine PE teachers’ job satisfaction and emotional exhaustion at work. Autonomously motivated PE teachers, who enjoy and value their teaching, will report greater job satisfaction, and will be better armed against emotional exhaustion, giving rise to better psychological functioning [1]. Several studies on PE teachers have offered support for this theoretical hypothesis, showing that autonomous motivation for teaching positively predicted job satisfaction [14,24], and negatively predicted emotional exhaustion [3,5]. On the other hand, controlled motivated PE teachers may put great effort into their teaching as well. However, these endeavors are mainly based on internal and external reasons. In this sense, although this type of PE teacher may not necessarily devote little effort to their work, their feelings of pressure may trigger maladaptive psychological functioning. Past studies on PE teachers have demonstrated that controlled motivation positively explained emotional exhaustion [3,5], and the associations with different positive work-related outcomes yielded mixed results (i.e., positive, negative, or non-significant) in school teachers (e.g., commitment, job satisfaction) [25,26] and PE teachers (e.g., need-supportive teaching style, vitality) [5,6]. Unlike autonomously and controlled motivated PE teachers, amotivated PE teachers do not find valuable reasons for engaging in their teaching. Furthermore, amotivation mostly arises from a low perceived satisfaction of teachers’ basic psychological needs (i.e., autonomy, competence, and relatedness), which, together, may trigger the worst pattern of psychological functioning [2]. One study on PE teachers showed a positive association between amotivation and emotional exhaustion [5]. Likewise, other studies among school teachers have shown a negative association between amotivation and job satisfaction [26].

### 1.4. What Value Does the Person-Centered Perspective Add to This Existing Evidence?

Although a variable-centered approach has been widely used to identify the link between different forms of motivation, and PE teachers’ job satisfaction and emotional exhaustion, this perspective does not consider the multidimensional nature of motivation [27]. On the contrary, a person-centered approach makes it possible to identify the multiple reasons teachers can combine to engage in their teaching. Two previous studies on PE teachers have shown that these professionals may combine autonomous and controlled forms of motivation when performing their jobs [3,6]. Four groups of PE teachers were found. A first group of PE teachers predominantly taught because they enjoyed and valued their jobs, while a second group basically only taught because they felt internally or externally pressured to do so [3,6]. Additionally, Van den Berghe et al. [3,6] found a third type of PE teachers who, although they felt internally or externally pressured, simultaneously took pleasure in their jobs, and identified with the value of the job. Finally, both studies identified a fourth group of PE teachers who did not perceive autonomous or controlled reasons to put effort into their teaching [3,6]. Even though these two studies add important contributions to our knowledge of PE teachers, no person-centered research to date has included the amotivation dimension in a sample of PE teachers. In the educational context, the additional value of amotivation has recently been explored by a study among secondary school teachers [28]. Consistent with Van den Berghe et al. [3,6], the study of Abós et al. [28] identified three distinct groups of autonomously motivated, controlled motivated, and combined autonomously-controlled motivated teachers. In addition, they found a group of teachers primarily characterized by moderate feelings of amotivation, combined with a few controlled reasons to do their jobs. Analogously, this “amotivation” group was found in a recent person-centered study developed with two samples of workers from different industrial sectors [27].

On the other hand, unlike a variable-centered approach, a person-centered approach allows the different forms of motivation to interact in predicting outcomes. According to SDT, the presence of a higher amount of motivation should not necessarily be better if this additional motivation is of poor quality (i.e., controlled motivation) [19]. Nevertheless, this theoretical hypothesis was only partially confirmed in previous studies on PE teachers [6]. Whereas no differences were found between the autonomous motivation group and the autonomous-controlled motivation group, in terms of emotional exhaustion [3,6], the autonomously motivated group reported higher need satisfaction values than the combined autonomous-controlled group [6]. Likewise, the results shown by Abós et al. [28] in secondary school teachers were not conclusive regarding whether the quality of motivation matters in terms of teachers’ psychological functioning.

In addition, also in line with SDT, PE teachers with high levels of amotivation and/or controlled motivation would report opposite results to the predominantly autonomously motivated group, showing the most maladaptive pattern of outcomes. This SDT-based hypothesis has been confirmed in PE teachers, showing that the relatively controlled motivated group displayed the highest levels of emotional exhaustion compared to the other three groups (i.e., autonomous motivation, combined autonomous-controlled motivation and low motivation groups) [3,6]. Furthermore, because PE teachers who display controlled reasons for teaching still have some desire to exert effort, SDT also suggests that amotivated PE teachers will experience more detrimental outcomes than controlled motivated teachers. Although this assumption has been confirmed in terms of need satisfaction, burnout, and engagement among secondary school teachers [28], it has not yet been specifically examined in PE teachers.

Finally, SDT also postulates that it would be more adaptive to display low levels of autonomous and controlled motivation, as opposed to displaying highly controlled motivation. This is because, while controlled motivation might trigger some positive outcomes (e.g., engagement), it simultaneously leads to highly detrimental outcomes for one’s psychological functioning, such as burnout [28,29]. In fact, this issue has been empirically demonstrated in previous studies among PE teachers, in which the group that was low on autonomous-controlled motivation reported lower levels of burnout than the relatively controlled group [3,6]. Moreover, there are no studies to date on PE teachers that have compared the possible differences between the low motivation group and the high amotivation group in terms of psychological functioning. Nevertheless, according to SDT, the group characterized by high levels of amotivation should always exhibit the most maladaptive pattern of outcomes [19].

### 1.5. The Present Study

Adopting a person-centered approach, the first aim of the study is to identify PE teachers’ motivational profiles based on their combinations of autonomous and controlled reasons to teach as well as the degree to which they are amotivated. According to SDT and past person-centered studies, we expect to find four or five distinct profiles. As in the two previous studies on PE teachers [3,6], we expect a “controlled motivated” group, a “not very motivated” group, a “combined controlled-autonomously motivation” group, and an “autonomously motivated” group to emerge. Furthermore, based on past studies on secondary school teachers [28] and workers [27], which also included the amotivation dimension, we anticipate that an additional group of PE teachers, characterized by high amotivation, would also emerge.

The present study aims to examine whether one motivational profile is more adaptive than another in terms of psychological functioning, by contrasting PE teachers’ scores on job satisfaction and emotional exhaustion within each of the retained profiles. Based on SDT and past person-centered studies [3,6,28], we hypothesize that the group predominantly characterized by high levels of autonomous motivation, as well as the group predominantly characterized by high levels of autonomous and controlled motivation, would display the highest levels of job satisfaction and the lowest values of emotional exhaustion. In contrast, we hypothesized that the groups characterized by the highest presence of controlled motivation or amotivation and absence of autonomous motivation would report the lowest job satisfaction and the highest emotional exhaustion. We expect the low motivation group to be in between. Relying on SDT, which suggests that the presence of more motivation is not necessarily better if it is of poor quality (i.e., controlled motivation), we further expect that the purely “autonomously motivated” group would display more job satisfaction and less emotional exhaustion than the “combined autonomous-controlled motivation” group. Given the findings of previous studies, which consistently display strong positive relationships between controlled motivation and emotional exhaustion [3,5], whereas mixed (i.e., positive, negative or null) relationships were noted between controlled motivation and job satisfaction [25,26], we expect the differences between these two groups to be larger for emotional exhaustion than for job satisfaction. Finally, we also expect that the “controlled motivation” group would display higher job satisfaction and lower emotional exhaustion than the “amotivation” group, because, according to SDT, the “controlled motivation” group still maintains some interest in doing their job.

## 2. Materials and Methods

### 2.1. Participants

This study has a cross-sectional design. A convenience sample of 107 in-service primary school PE teachers from the region of Aragon participated in the current study. In Spain, primary teachers teach students from six to 12 years old. Both genders were equally represented in the sample, resulting in 61 male (57%) and 46 (43%) female PE teachers. The average age of the participants was 34.87 (*SD* = 5.77) years old, representing a range of ages from 25 to 50 years old. Furthermore, they had been working as PE teachers for an average of 7.47 (*SD* = 5.63) years, ranging from one to 45 years. Most participants (82%) taught in state schools, although PE teachers from non-state schools were also represented (18%).

### 2.2. Procedures

Ethical approval for this study was obtained from the first author’s university research ethics committee (CEICA; PI15/0283). Participants were recruited via social media (i.e., Facebook and Twitter). More precisely, a PE teachers’ association (named “+ *Educación Física*”: www.masefaragon.com) from the region of Aragon collaborated in the study by asking primary school PE teachers to participate in the study via their social media profiles. This PE association was comprised of 150 primary school PE teachers at the time of recruitment. Those teachers interested in participating were asked to follow a weblink to a 28-item online questionnaire assessing sociodemographic characteristics (i.e., gender, age, teaching experience, and type of school), motivation for teaching, job satisfaction, and emotional exhaustion. The website provided an explanation of the aim and procedure of the study, asking for the teachers’ consent to participate in the study, and offering participants the opportunity to get in touch with the main researcher in the event of questions. The online questionnaire was pilot-tested to identify potential problems and to estimate completion time. The time required to complete and submit the questionnaire was 15 days. Participation was voluntary and the confidentiality of the PE teachers’ responses was guaranteed.

### 2.3. Measures

#### 2.3.1. PE Teachers’ Motivation for Teaching

Motivation was measured using the Spanish version of the Motivation for Teaching Scale [30]. This scale began with the stem, “I get involved in teaching because..”. followed by 19 items evaluating intrinsic motivation (four items; e.g., “I think teaching is a pleasant activity”), identified regulation (four items; e.g., “I believe that it is an important objective in my life”), introjected regulation (four items; e.g., “On the contrary, I would feel guilty”), external regulation (four items; e.g., “I feel forced to do so by others”) and amotivation (three items, e.g., “I do not know, I feel like I am wasting time when I teach”). To meet the aim of this study and for parsimony reasons, analyses were conducted using composite scores for autonomous and controlled motivation. The calculation of composite scores is a widely used procedure in the SDT framework, and has also been used in other studies that assess PE teachers’ motivation [6]. Responses were given on a five-point Likert scale ranging from 1 (“strongly disagree”) to 5 (“strongly agree”). Cronbach’s alpha was 0.75, 0.74, and 0.72 (i.e., acceptable) [31] for autonomous motivation, controlled motivation, and amotivation, respectively.

#### 2.3.2. PE Teachers’ Job Satisfaction

Job satisfaction was measured using a Spanish version of the Teacher Job Satisfaction Scale [20]. Evidence of reliability has been shown for this four-item scale in prior research in Spanish teachers [32]. Example items are “Working as a teacher is extremely rewarding” and “I look forward to going to school every day”. Responses were given on a six-point Likert scale from 1 (“strongly disagree”) to 6 (“strongly agree”). Cronbach’s alpha was 0.83 (i.e., good) [31].

#### 2.3.3. PE Teachers’ Emotional Exhaustion

Emotional exhaustion was measured using the Spanish version of Maslach Burnout Inventory-General Survey [33]. This scale examines the burnout dimensions of emotional exhaustion, depersonalization, and reduced personal accomplishment. However, because emotional exhaustion is considered the archetype of burnout [22], and in line with other studies on PE teachers [34], only the five items assessing emotional exhaustion were used in the present study. Example items are, “I feel burned out from my work” and “I feel used up at the end of the workday”. Responses were reported on a seven-point Likert scale from 0 (“strongly disagree”) to 6 (“strongly agree”). Cronbach’s alpha was 0.86 (i.e., good) [31].

### 2.4. Data Analysis

Firstly, descriptive statistics, Pearson’s correlations, and Cronbach’s alpha reliabilities were calculated. Cronbach’s alpha values above 0.70 and 0.80 were considered acceptable and good, respectively [31]. PE teachers with values of more than three standard deviations above or below the mean, or with high Mahalanobis values, were removed to reduce the impact of univariate and multivariate outliers, respectively. Subsequently, cluster analyses were conducted using autonomous motivation, controlled motivation, and amotivation. A combination of hierarchical and non-hierarchical clustering methods was carried out in two consecutive steps. The first step consisted of conducting a hierarchical cluster analysis using Ward’s method based on squared Euclidean distances. Three to five cluster solutions were tested. To identify the number of cluster solutions, the percentage of explained variance and a dendrogram were visually inspected. Using the extracted initial cluster centers based on Ward’s hierarchical method as non-random starting points, the second step addressed an iterative, non-hierarchical k-means clustering procedure. The stability based on Cohen’s Kappa (values higher than 0.50 are considered acceptable) of the retained cluster solution was examined by using the double-split cross-validation procedure. The possibility of including sociodemographic variables (i.e., gender, age, teaching experience, and type of school) as covariates in subsequent analyses was considered and explored using the Chi-square test and multinomial regression. Finally, a multivariate analysis of variance (MANOVA) with post hoc tests using the Bonferroni method was conducted to examine differences between the retained clusters, and PE teachers’ job satisfaction and emotional exhaustion. Effect sizes (η_p_^2^) above 0.01 were considered small, above 0.06 moderate, and above 0.14 large [35]. All analyses were conducted using SPSS 20.0 software (IBM SPSS Inc., Chicago, IL, USA).

## 3. Results

Descriptive statistics for PE teachers’ responses to study variables and Pearson’s correlations are reported in Table 1.

Prior to conducting the cluster analysis, six outliers were removed (four univariate and two multivariate) resulting in a final sample of 101 PE teachers (56 males). Four different clusters were identified, explaining 51%, 62%, and 72% of the variance in autonomous motivation, controlled motivation, and amotivation, respectively. A three-cluster solution explained too little variance in autonomous motivation (i.e., 30%), thereby not reaching the critical threshold of 50% [36]. A five-cluster solution was not chosen because it was difficult to interpret, and some of the clusters represented less than 5% of the study sample [37]. For the retained four-cluster solution, the double-split cross-validation procedure provided an average kappa value of 0.70, indicating good stability.

**Figure 1:**
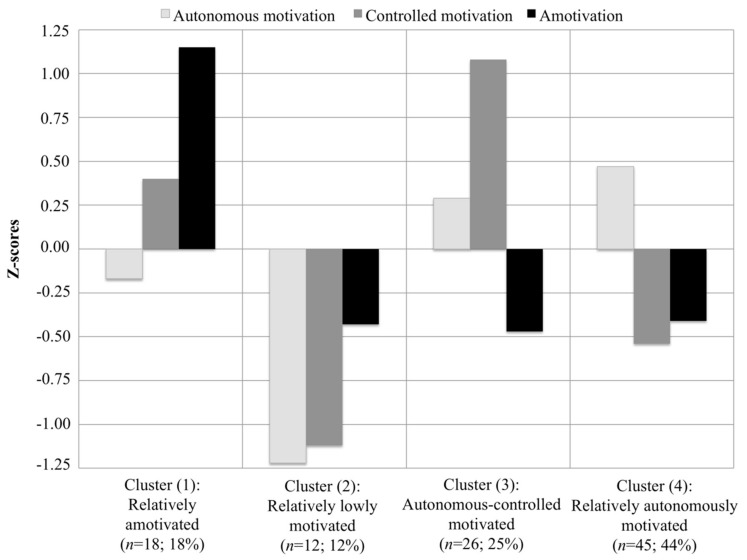
Four-cluster solution based on standardized scores for autonomous motivation, controlled motivation, and amotivation for primary PE teachers.

The graphical results for the four-cluster solution based on *Z*-scores (Y-axis) with regard to autonomous motivation, controlled motivation, and amotivation are presented in Figure 1. From left to right, the four clusters were characterized and labelled as follows. The “relatively amotivated” group displayed the highest levels of amotivation, together with relatively low levels of autonomous motivation, and low to moderate levels of controlled motivation. The “relatively low motivation” group showed the lowest levels of autonomous and controlled motivation, accompanied by very low levels of amotivation. The “autonomous-controlled motivated” group displayed the highest levels of controlled motivation, along with high levels of autonomous motivation and very low levels of amotivation. Finally, the “relatively autonomously motivated” group showed the highest levels of autonomous motivation, low levels of controlled motivation, and very low levels of amotivation.

The standardized and absolute scores, and Bonferroni pairwise comparisons in the four clusters, in autonomous motivation, controlled motivation, and amotivation, are presented in Table 2. Next, prior to conducting a MANOVA including our two outcome variables, we examined the cluster assignment by sociodemographic variables. Chi-square testing revealed a non-significant cluster assignment by gender (χ^2^[3, *n* = 101] = 2.76, *p* > 0.05) and type of school (χ^2^[3, *n* = 101] = 0.75, *p* > 0.05). In addition, multinomial regression analysis revealed no association between age (Pseudo-*R*^2^ Nagelkerke = 0.53, *p* > 0.05) and teaching experience (Pseudo-*R*^2^ Nagelkerke = 0.44, *p* > 0.05) with the four-cluster solution. Based on these results, none of the sociodemographic variables were considered as covariates in subsequent analyses.

**Figure 2:**
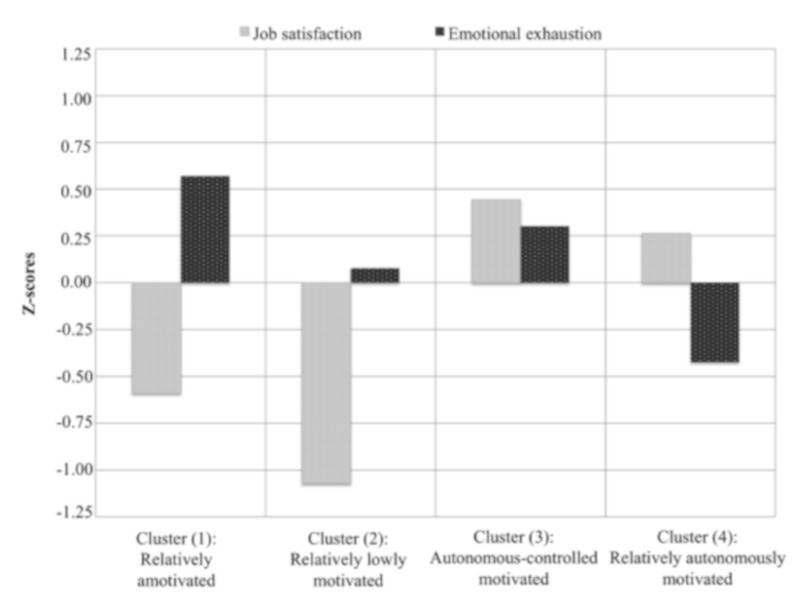
Standardized job satisfaction and emotional exhaustion scores in the retained four-cluster solution.

The last step was to examine differences in PE teachers’ work-related outcomes, according to the retained motivational groups. Using the four clusters as the independent variable, MANOVA showed a significant multivariate effect on study variables with a high effect size (*F*(15, 275.13) = 34.64, *p* < 0.001, η_p_^2^ = 0.63). Bonferroni pairwise comparisons between clusters were performed. *F*-values and univariate effect sizes (η_p_^2^) are reported at the bottom of Table 2. With regard to job satisfaction, the highest values were found in the “autonomous-controlled motivated” group and the “relatively autonomously motivated” group. These two high autonomous motivation groups revealed significantly higher levels of job satisfaction than the “relatively low motivation” group and the “relatively amotivated” group. No differences in job satisfaction were found between the “relatively low motivation” group and the “relatively amotivated” group. As regards emotional exhaustion, the “relatively autonomously motivated” group reported significantly lower levels than the other three groups, with the exception of the “relatively low motivation” group with which no differences were found. Figure 2 graphically shows the standardized scores of job satisfaction and emotional exhaustion according to the four clusters retained.

## 4. Discussions

Within the last decade, emergent research in PE teachers has shown how these professionals can strive in their teaching, due, simultaneously, to autonomous and controlled reasons [3,6]. However, no person-centered studies to date have included the three types of motivation proposed by SDT (i.e., autonomous motivation, controlled motivation, and amotivation) using cluster analysis. This issue seems even more interesting if we consider the numerous difficulties and stressors inherent in PE teaching [14]. To fill this gap, this study aimed to identify which combinations of autonomous motivation, controlled motivation, and amotivation in PE teachers naturally coexist, and how these motivational groups may differ in terms of job satisfaction and emotional exhaustion.

With regards to the first aim, and consistent with our hypothesis, results of the cluster analyses pointed towards the retention of four significantly distinct groups of PE teachers in terms of motivation to teach. In an absolute sense, the study sample reported a very high average of autonomous motivation (*M* = 4.52/5), a moderate average of controlled motivation (*M* = 2.91/5), and a very low average of amotivation (*M* = 1.16/5). Apparently, PE teachers from the present study predominantly experienced autonomous reasons for teaching and were scarcely amotivated. Indeed, even in the “relatively amotivated” group, absolute scores for autonomous motivation were still rather high, and absolute scores for amotivation were still rather low. Given the large amount of literature that warns against the low level of recognition and the low value that PE teachers perceive in terms of their subject [9,34], the “high autonomous motivation” group found in these PE teachers may be interpreted as a piece of hopeful information for their psychological functioning. In any case, before continuing, it should be noted that the terms “relatively”, “high”, and “low” refer to scores that are compared with the scores of other PE teachers in this study. The labeling of the four motivational groups, therefore, should be considered a matter of gradation.

In line with previous research among PE teachers [3,6], and consistent with our hypothesis, a “relatively autonomously motivated” group emerged, accounting for 44% of the sample. As occurred in the two previous studies on PE teachers [3,6], this group was the most representative of the sample (i.e., 44%). In this sense, almost half of the PE teachers in this sample barely put any effort into their work for controlled or unregulated reasons, as they are highly autonomously motivated. In fact, in Spain, it is compulsory to pass a very complicated national exam to become a PE teacher [15]. So, given the effort and dedication required to prepare for this test, it is possible that PE teachers who pass are characterized by mainly autonomous reasons for teaching, which could explain the results. Furthermore, consistent with past studies [3,6] and as expected, we identified a combined “autonomous-controlled motivated” group accounting for 25% of the participants. This motivational group indicates that at least one quarter of the PE teachers of this study combine both autonomous and controlled reasons to engage in teaching. In this sense, it is important to note that some PE teachers who enjoy teaching can also perceive other reasons for putting effort into their teaching, such as economical compensation or social approval. Moreover, a “relatively low motivation” group emerged, which captured the lowest number of PE teachers (12% of the sample). Although the two previous studies among PE teachers only included the dimensions of autonomous and controlled motivation [3,6], they also found a similar “low motivation” group. However, in a previous study in secondary school teachers, which included the three types of motivation, this profile was not identified [28]. It seems that PE teachers, therefore, might combine moderate and low autonomous and controlled reasons, respectively, to engage in teaching, together with very low feelings of amotivation. Nevertheless, more research is required to fully confirm this type of profile within PE teachers. Finally, a “relatively amotivated” group was identified accounting for 18% of the PE teachers. Although previous studies on PE teachers did not include the amotivation dimension in their cluster analysis, this fourth group has also been identified in studies among secondary school teachers [28] and workers [27]. Consequently, it seems that, while PE teachers cannot combine autonomous and amotivated reasons for teaching, they could have relatively high feelings of amotivation along with some controlled reasons for teaching.

Job satisfaction is crucial to reach good psychological functioning among PE teachers, which may also have an impact on PE teacher attrition/retention rates [14], and on the students’ academic achievement [38]. In this sense, the results associated with the second aim revealed that the “relatively autonomously motivated” group and the “autonomous-controlled motivated” group displayed significantly higher scores on job satisfaction compared to the “relatively low motivation” group and the “relatively amotivated” group. As such, it is the presence of autonomous motivation, either in the presence or absence of controlled motivation, that affects job satisfaction [19]. The presence of controlled motivation in itself (without autonomous motivation) does not suffice to develop the highest levels of job satisfaction. Logically, while experiencing feelings of pressure may be valid in terms of productivity, in line with SDT [19], it seems complicated that controlled motivation, by itself, may be associated with feelings of job satisfaction. In addition, no differences in terms of PE teacher job satisfaction were found between the “relatively autonomously motivated” group and the combined “autonomously-controlled motivated” group. This suggests that the addition of controlled motivation to autonomous motivation does not hamper teachers from experiencing job satisfaction. Said differently, if teachers enjoy and value their jobs, they experience job satisfaction, and this is independent of whether they also feel externally or internally pressured to engage in teaching. These results are aligned with past research in secondary school teachers and workers [27,28]. In these two studies, the autonomously motivated group and the combined autonomous-controlled group exhibited the highest engagement and job satisfaction values, while hardly any significant differences were found between these two profiles on these study variables. Turning our attention to emotional exhaustion, a risk factor for multiple maladaptive outcomes in teachers [39], the “relatively autonomously motivated” group reported the most adaptive pattern, except for the “relatively low motivation” group, in which no differences were found. Indeed, consistent with previous person-centered studies [27,28] and in line with SDT, it was the “relatively autonomously motivated” group that experienced the lowest levels of emotional exhaustion. Finally, contrary to our expectations, the “autonomous-controlled motivated” group did not experience less emotional exhaustion when compared to the “relatively low motivation” group or the “relatively amotivated” group.

Overall, these results suggest that the quality of motivation matters for PE teachers’ job satisfaction, and more in particular, for their feelings of emotional exhaustion. These findings are even more noteworthy if we consider that the “relatively autonomously motivated” and “autonomous-controlled motivated” groups were analogous in terms of autonomous motivation and amotivation, and the only difference between the two groups were the higher levels of controlled motivation displayed by the “autonomous-controlled motivated” group. Up until this study, previous research in PE teachers had shown that the predominantly autonomously motivated group displayed the lowest levels of emotional exhaustion [3,6]. However, no significant differences in terms of emotional exhaustion were found between the “autonomous motivation” group and the “combined autonomous-controlled motivation” group in either of the two studies. Although we have to be cautious, our results may be considered as a remarkable contribution. In this sense, according to SDT’s qualitative view of motivation [19], more motivation is not necessarily better in terms of emotional exhaustion among PE teachers if this motivation is less self-determined.

Our last hypothesis stated that the “controlled motivation” group would display higher job satisfaction and lower emotional exhaustion than the “relatively amotivated” group. However, of these two postulated profiles, only the “relatively amotivated” group emerged. Nevertheless, another interesting comparison with the “relatively low motivation” group may be discussed. The “relatively amotivated” group displayed higher levels in the three dimensions of motivation (i.e., autonomous, controlled, and amotivation) than the “relatively low motivation” group. Considering these differences, the “relatively amotivated” group could be expected to show lower job satisfaction and higher emotional exhaustion, especially because of the higher levels of amotivation [19]. However, contrary to our expectations, neither of the groups differed from each other in either of the two psychological outcomes in the present study. Based on this result, it seems that although PE teachers experience slightly lower levels autonomous motivation (i.e., as in the case of the “relatively low motivation” group), their psychological functioning will be similar if they also experience lower levels of controlled motivation and amotivation [1,19]. This evidence notably emphasizes that although autonomous reasons are crucial for adequate development and personal growth, reducing feelings of controlled motivation and amotivation as much as possible can be equally or even more important.

### 4.1. Suggestions for Practice

When we talk about creating a motivating school climate, this is not only about learners. Teachers themselves are a decisive element in the classroom [40,41]. In addition, teachers bring their motivation to the classroom, and, there, motivation is important for their job satisfaction and to avoid emotional exhaustion [38]. Some suggestions resulting from the present study, which may be useful in practice, are discussed. Results suggest that PE teachers who score moderate on controlled motivation and low to moderate on amotivation are more likely to experience emotional exhaustion. This means that it is fundamental to assess PE teachers’ motivation and to intervene by applying specific and effective strategies whose aim is to reach more self-determined forms of motivation, but especially to avoid controlled motivation and amotivation. PE teachers are more likely to engage in teaching for autonomous reasons when their three basic psychological needs of autonomy, competence, and relatedness are satisfied [2]. However, at schools, teachers’ autonomous motivation is too often hampered because their basic psychological needs for autonomy (i.e., feeling that oneself is the causal agent of one’s actions), competence (i.e., perceiving ability with a situation that threatens an important goal), and relatedness (i.e., experiencing social inclusion and warm interpersonal relationships) are undermined by administrative control, inflexible curricula or lack of support [2,16]. During the past decade, more and more SDT researchers have examined the contextual factors that influence teachers’ need satisfaction and their autonomous motivation. Evidence shows that when teachers are empowered, and receive confidence and opportunities to be creative, they will benefit [3,6,28,32,42]. Just as teachers face the challenge of motivating their learners, policy makers and principals are thus challenged to motivate teachers in their jobs [3,6].

### 4.2. Limitations and Future Research Directions

The present study contains certain limitations that should be considered when interpreting the results. First, the results of this research were derived from cross-sectional data, which makes it impossible to investigate whether PE teachers’ motivation for teaching changes over time, and how these variations affect their psychological functioning. Given there is evidence that PE teachers’ motivation is a variable that might fluctuate due to changes in an individual’s social and economic environment [16], more longitudinal and daily-diary research seems required to shed light on this gap. Another limitation that should be recognized is the use of a non-probabilistic sampling method to recruit the participants. Future studies could try to replicate the study by testing the hypotheses with a larger and more representative sample of PE teachers. Third, another feature of the present study was that PE teachers were only recruited through social media. One of the problems is that teachers who interact in social media groups may be more likely to be highly autonomously motivated for teaching. Even though this recruitment method is common in social science research, it may lead to self-selection bias, and therefore may impact the ability to make generalizations about the study results. To overcome this limitation, future research could combine this type of recruitment with other traditional methods (i.e., web, telephone or paper and pencil surveys). In addition, the use of qualitative techniques could contribute a more detailed analysis of reasons that lead PE teachers to experience different motivational patterns. Finally, in this study, only two psychological outcomes (i.e., job satisfaction and emotional exhaustion) of PE teachers were explored. Replicating and expanding this research by adding antecedents of PE teachers’ motivation (e.g., need satisfaction, need frustration, and need dissatisfaction), and other relevant PE teaching-related outcomes (e.g., interpersonal teaching style or engagement), should be considered as a new avenue of research.

## 5. Conclusions

By using a person-centered approach, this research provides new evidence about how PE teachers may combine qualitatively different reasons for teaching. The most adaptive motivational profile in terms of psychological functioning is characterized by high autonomous motivation, low to moderate controlled motivation, and low amotivation. Identified as the most adaptive profile, this purely “autonomously motivated” group seems beneficial for PE teachers’ job satisfaction, and, moreover, seems to be especially crucial to buffer feelings of emotional exhaustion. In fact, this predominantly autonomous group is less likely to experience emotional exhaustion than the group that combines high autonomous motivation with moderate to high controlled motivation. In sum, the present study offers support for SDT’s qualitative point of view on motivation. Albeit, these results suggest that the quality of motivation matters in terms of emotional exhaustion, some differences were also found in terms of job satisfaction. These findings suggest that more motivation is not necessarily better among PE teachers in terms of psychological functioning when this additional motivation arises from controlled reasons.

## Figures and Tables

**Table 1:** Range, means, standard deviation, and Pearson’s correlations among the study variables.

Study Variables	Range	*M* (*SD*)	1	2	3	4	5	6	7
1. Autonomous motivation	1–5	4.52 (0.37)	−						
2. Controlled motivation	1–5	2.91 (0.67)	0.25 *	−					
3. Amotivation	1–5	1.16 (0.25)	−0.28 **	0.13	−				
4. Job satisfaction	1–6	4.68 (0.73)	0.51 **	0.24 *	−0.30 **	−			
5. Emotional exhaustion	0–6	1.58 (1.10)	−0.10	0.22 *	0.30 **	−0.18 *	−		
6. Age	25–50	34.87 (5.77)	−0.08	0.01	0.06	−0.11	0.01	−	
7. Teaching experience	1–45	7.47 (5.63)	0.03	−.05	−0.08	−0.11	0.01	0.72 **	−

Note: * = *p* < 0.05; ** = *p* < 0.01.

**Table 2:** Motivational cluster mean scores, *F*-values, and effect sizes (η_p_^2^) for PE teachers’ motivation, job satisfaction, and emotional exhaustion.

Study Variables	Cluster (1): Rel. Amotivated	Cluster (2): Rel. Low Motivation	Cluster (3): Autonomous-Controlled Motivated	Cluster (4): Rel. Autonomously Motivated		
	*n* = 18 (18%)	*n* = 12 (12%)	*n* = 26 (25%)	*n* = 45 (44%)	*F* ^(3,97)^	η_p_^2^
**Autonomous motivation**						
*Z*-scores	−0.17 (0.15) ^2,4^	−1.22 (0.19) ^1,3,4^	0.29 (0.13) ^2^	0.47(0.09) ^1,2^	22.64 *	0.41
Absolute scores (1–5)	4.52 (0.06) ^2,4^	4.05 (0.08) ^1,3,4^	4.72 (0.05) ^2^	4.80 (.04) ^1,2^		
**Controlled motivation**						
*Z*-scores	0.40 (0.14) ^2,3,4^	−1.12 (0.18) ^1,3,4^	1.08 (0.12) ^1,2,4^	−0.54 (0.09) ^1,2,3^	51.34 *	0.61
Absolute scores (1–5)	3.21 (0.10) ^2,3,4^	2.18 (0.12) ^1,3,4^	3.67 (0.08) ^1,2,4^	2.58 (0.06) ^1,2,3^		
**Amotivation**						
*Z*-scores	1.15 (0.08) ^2,3,4^	−0.43 (0.10) ^1^	−0.47 (0.06) ^1^	−0.41 (0.05) ^1^	99.13 *	0.75
Absolute scores (1–5)	1.61 (0.03) ^2,3,4^	1.02 (0.02) ^1^	1.01 (0.03) ^1^	1.03 (0.01)^1^		
**PE teachers’ psychological functioning**						
Job satisfaction (1–6)	4.25 (.17) ^3,4^	3.89 (0.20) ^3,4^	5.00 (0.10) ^1,2^	4.87 (0.13) ^1,2^	12.77 *	0.28
Emotional exhaustion (0–6)	2.21 (0.17) ^4^	1.66 (0.19)	1.91 (0.10) ^4^	1.11 (0.13) ^1,3^	6.34 *	0.16

Note: Rel. = Relatively; Standard errors are reported in parentheses. Numbers in superscript (1 to 4) refer to significantly different groups. Differences between the four clusters were inspected, repeating the equations twice and modifying the reference category. So, coefficients for each group were extracted, allowing pairwise comparisons. * = *p* < 0.001.
